# Preparing Medical and Nursing Students for Interprofessional Feedback Dialogues

**DOI:** 10.5334/pme.1069

**Published:** 2023-10-31

**Authors:** Claudia Tielemans, Renske de Kleijn, Emy van der Valk - Bouman, Sjoukje van den Broek, Marieke van der Schaaf

**Affiliations:** 1 Education Centre, Unit of Medical Education, of the University Medical Centre Utrecht, Utrecht, NL; 2 Utrecht Center for Research and Development of Education of the University Medical Centre Utrecht, Utrecht, NL; 3 Utrecht University when the research was conducted. Currently she is a PhD student at Erasmus Medical Centre, Rotterdam, NL; 4 Department Clinical Skills Training of the University Medical Centre Utrecht, Utrecht, NL; 5 Utrecht Center for Research and Development of Education at University Medical Centre Utrecht, Utrecht, NL

## Abstract

**Background:**

In healthcare education, preparing students for interprofessional feedback dialogues is vital. However, guidance regarding developing interprofessional feedback training programs is sparse. In response to this gap, the Westerveld framework, which offers principles for interprofessional feedback dialogue, was developed.

**Approach:**

Using the Westerveld framework, we developed and implemented an interprofessional feedback intervention for 4^th^-year nursing and 5^th^-year medical students. It encompasses two half-day workshops comprising small group sessions, interactive lectures, and a goal-setting assignment for the rotations. This paper describes the intervention and reflects on students’ self-reported goals, as learning outcomes, to inform future interprofessional feedback dialogue education.

**Outcomes:**

To understand student’s learning outcomes, we coded the content and specificity of 288 responses to the goal-setting assignment. Students indicated they mainly aimed to improve their feedback actionability, but contrastingly set – largely unspecific – goals, addressing the initiation of feedback dialogues. To better understand the process of setting these goals, we held three focus groups (N = 11): aside from the Westerveld framework, students used previous experience in rotations, outcome expectations, and personal characteristics as sources in their goal-setting process.

**Reflection:**

The contrast between students’ aims to improve their actionability and their goals to initiate dialogues, suggests that overcoming practice barriers to initiating dialogues are conditional to developing other feedback dialogue aspects. These and other goal conflicts in the workplace may hinder them setting specific feedback dialogue goals. We recommend explicit discussion of these challenges and conflicts in interprofessional feedback dialogue education.

## Background and need for Innovation

Healthcare professionals increasingly need to collaborate interprofessionally [1,2,3]. Within these interprofessional collaborations, feedback dialogues are essential, which involve professionals actively seeking, giving, sharing, and discussing feedback information [4]. We hereby follow the current trend in higher education to define feedback as (communicative) process, rather than as information [5]. Defined in the interprofessional context these dialogues are held by ‘members of two or more professions’ and are ‘about individual or team performance’ [6, p1]. Though preparing students for interprofessional feedback dialogues is a well-established aim for healthcare education, guidance for developing interprofessional feedback training has been sparse [6,7,8,9,10].

## Goal of Innovation

**Figure 1:**
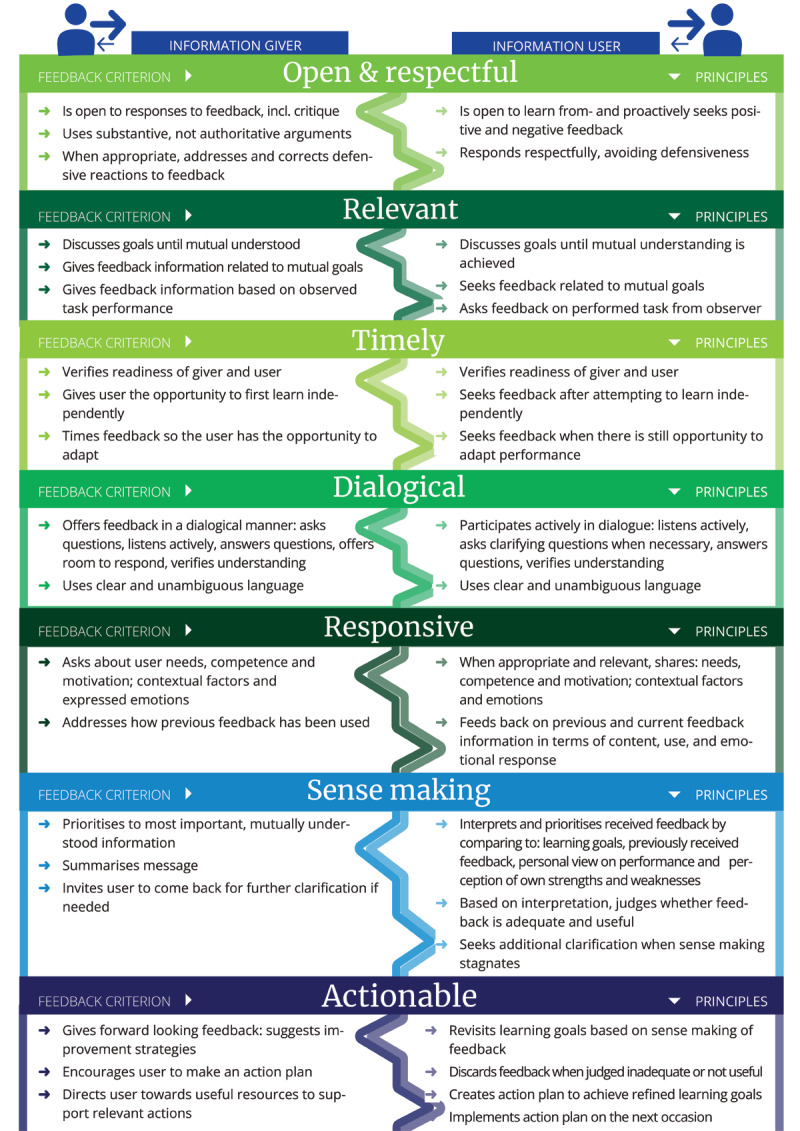
Summary of the Westerveld framework for interprofessional feedback dialogues [4].

Therefore, we developed and implemented an intervention aimed at enhancing the feedback-giving and -receiving skills of nursing and medical students in interprofessional workplace dialogues. The intervention was based on the Westerveld Framework for Interprofessional Feedback Dialogues (WVF; for a summary visual see Figure 1). This framework was developed through a critical literature review and by an international expert panel [4]. The WVF comprises seven criteria to describe the principles of interprofessional feedback dialogue: *Open and Respectful, Relevant, Timely, Dialogical, Responsive, Sense making, Actionable*. The framework has two distinctive features: a) it is the first to integrate giving *and* using feedback information in one framework, as healthcare professionals are expected to take both these feedback roles and b) it describes how to recognize and address interprofessional context barriers in feedback dialogues.

The overarching aim of the innovation was for students to reflect on complex interprofessional feedback dialogues and set individual learning goals to further improve their interprofessional feedback dialogue skills. We specifically chose to focus on students setting individual learning goals, as this would require them to relate the content of the WVF to their own (interprofessional) rotation experiences and their views on what they already have or have not mastered yet. Goal setting is a powerful way to direct performance and manage learning in training, as intention is considered an important step towards behavioral change and intentional learning [11,12]. So, goal-setting requires a meaningful connection between the WVF and students’ experiences. With the goal-setting assignment, we aimed to converge their attention and focus for their next rotation towards a specific element they wanted to improve on.

## Steps taken for Development and Implementation of Innovation

### Timing and placement in curricula

We designed and piloted the Westerveld Interprofessional Feedback Intervention (WIFI) during the 2019–2020 academic year and have since iteratively refined its design. WIFI is mandatory for all 4^th^ year nursing students (in a four-year curriculum) and 5^th^ year medical students (in a six-year curriculum) in a medical and nursing school in the Netherlands. This placement in their curricula was selected because all students: a) had at least one year of clinical-rotation experience, which included interprofessional collaboration, to inform their participation in the intervention; b) would immediately, or soon after the classroom sessions return to practice, where they could apply what they had learned; and c) had experience with reflection, self-assessment, and goal-setting from previous years of training.

### Overall structure

Approximately 100 students (30% medicine, 70% nursing) participated in WIFI every six weeks. WIFI was a classroom-based intervention consisting of two half-day workshops, one week apart, both containing two elements: a 1.5-hour small group session and a 1-hour interactive lecture. Both were taught by healthcare professionals.

### Interactive lectures

The interactive lectures were held with approximately 50 mixed medical and nursing students. In the first lecture, the students familiarized themselves with the WVF. Students discussed written feedback dialogue examples based on real scenarios from practice, using the framework. Reflective prompts were: *On what criteria does this example do well? Why? On what criteria could it improve? How? Which example do you prefer? Why?*

In the second lecture, students watched video-examples in which unsought interprofessional feedback information had to be given. In plenary discussions they then used the interprofessional additions of the WVF to address interprofessional context barriers. Reflective prompts were: *Would you speak up? Why? Would speaking up be easier in the same situation with a monoprofessional colleague? How could you approach such a situation?*

### Small group sessions

The small group sessions consisted of eight medical and nursing students. Divided over the two sessions, students worked on three cases, which were designed to help students discover each other’s perspectives, based on real scenarios from practice. Medical and nursing students mainly received the same information, with a different nuance based on their professional perspective. For example, medical students received information about a medically discharge-ready patient and the ward’s need to empty beds for new patients, whilst nursing students received information about the home-situation and impaired mobility of that patient.

In the second session, the third case was followed by a feedback simulation exercise. Two students role-played a feedback dialogue based on a complex interprofessional workplace-situation. The other students observed using the WVF, and, guided by the teacher, provided peer-feedback information on the simulated dialogue on 1 or 2 criteria per observing student.

At the end of WIFI, students were asked to individually articulate their learning goals for feedback dialogues in their next rotation. Three guiding questions in this goal-setting assignment were: a) *In which feedback role can you still learn the most: as feedback information giver or receiver? b) On what Westerveld criterion do you aim to improve in the next rotation? c) Please set a goal for your next rotation regarding interprofessional feedback*. Students could voluntarily enter their answers in a digital form for research purposes.

Ethical approval was gained from the Dutch Association for Medical Education (NVMO), file number 2021.7.1. Goal-setting assignments were anonymously abstracted from the ELO and all focus group participants signed informed consents prior to participation.

## Outcomes of Innovation

### Goal-setting assignment

To understand students’ intention when returning to clinical practice, we analyzed their goal-setting assignments in the 2020–2021 academic year cohorts. 288 out of 1069 students volunteered their answers anonymously. This response rate of 27% is lower than our generally encountered 30–35%. First, we looked at the frequencies of students’ answers to: the Feedback role they wanted to improve in, and the Westerveld criterion they wanted to improve on (questions a and b of the goal-setting assignment). Second, we coded the goals students subsequently set (question c) deductively on the seven criteria of the WVF. For example, “*To ask for clarification where necessary”* was coded as Dialogical, and “*To express my own opinion and experience”* was coded as Adaptive. Third, using a rating scheme adapted from Hanley et al. [13], we coded the goals on level of specificity as Good, Fair, or Poor. Goals were coded independently by EB and CT. Discrepancies were resolved by consensus discussion. See Table 1 for the results of this analysis.

**Table 1:** Students’ goal setting assignment answers and goal content.


*QUESTION A) IN WHICH FEEDBACK ROLE CAN YOU STILL LEARN THE MOST?*			

ANSWER	ALL STUDENTS N (%)	NURSING N (%)	MEDICINE N (%)

As feedback information giver	198 (69%)	100 (76%)	44 (56%)

As feedback information receiver	84 (29%)	28 (21%)	32 (41%)

Missing	6 (2%)	4 (3%)	2 (3%)

**Total**	**288 (100%)**	132* (100%)	78*(100%)

***QUESTION B) ON WHAT WESTERVELD CRITERION DO YOU AIM TO IMPROVE IN THE NEXT ROTATION?***			

Open and Respectful	11 (5%)	7 (5%)	4 (5%)

Relevant	8 (4%)	6 (5%)	2 (3%)

Timely	32 (15%)	20 (15%)	11 (14%)

Dialogical	26 (12%)	20 (15%)	6 (8%)

Responsive	27 (13%)	12 (9%)	14 (18%)

Sense making	16 (8%)	11 (8%)	5 (6%)

Actionable	56 (26%)	34 (26%)	22 (28%)

Missing	36 (17%)	22 (17%)	14 (18%)

**Total**	212** (100%)	132* (100%)	78* (100%)

***QUESTION C) PLEASE SET A LEARNING GOAL FOR YOUR NEXT ROTATION REGARDING INTERPROFESSIONAL FEEDBACK***.			

**Goal code**			

*criterion*			

Open and Respectful	75 (26%)	32 (24%)	14 (18%)

Relevant	17 (6%)	7 (5%)	6 (8%)

Timely	25 (9%)	10 (8%)	10 (13%)

Dialogical	11 (4%)	5 (4%)	4 (5%)

Responsive	15 (5%)	8 (6%)	3 (4%)

Sense making	6 (2%)	0 (0%)	1 (1%)

Actionable	12 (4%)	6 (5%)	4 (5%)

No feedback goal	24 (8%)	10 (8%)	10 (13%)

Missing	103 (36%)	54 (41%)	26 (33%)

**Total**	**288 (100%)**	132* (100%)	78* (100%)

*specificity*			

Poor	134 (46%)	58 (44%)	35 (45%)

Fair	47 (16%)	18 (14%)	15 (19%)

Good	4 (1%)	2 (2%)	2 (3%)

Missing	103 (36%)	54 (41%)	26 (33%)

**Total**	**288 (100%)**	132* (100%)	78* (100%)


* Study program (Nursing/Medicine) was not asked in the first two cohorts (n = 76) and missing for two more students (n = 2). ** Question b was not asked in the first two cohorts (n = 76).

Lastly, we used Pearson’s Chi-Square analysis to estimate associations between Profession and: Feedback role, Westerveld criterion intended to improve on, and Westerveld criterion most addressed by goal. The data on Specificity were too skewed to analyse. To determine effect sizes, we calculated Phi for Feedback role, and Cramer’s V for the other two variables. IBM SPSS Statistics (Version 26.0) was used for statistical analyses. The Feedback role in which students wanted to improve was statistically significantly associated with profession (medicine or nursing), χ^2^ (1,N = 204) = 9.40, p < 0.01. Indicating, with a small-moderate effect size, Phi was 0.22, that nursing students were more likely to want to improve as feedback information givers than medical students. The criterion on which students wanted to improve was not statistically significantly associated with profession, χ^2^ (7,N = 204) = 6.30, p = 0.51, Cramer’s V was 0.18, as was the criterion addressed by students’ goals, χ^2^ (8,N = 204) = 7.26, p = 0.51, Cramer’s V was 0.19.

As we found that most goals were coded as Open and Respectful (n = 75), we inductively created six sub-codes for this criterion: *giving* feedback information (36%), being *assertive* (28% e.g., “*To stand up for myself and to dare to start dialogues with doctors”*), being *open* (20%), *asking* for feedback information (11% e.g., “*I am going to ask more feedback from other professionals”*), being *respectful* (3%), and *receiving* feedback information (3%). Except for being more respectful, all these categories addressed the initiation of a feedback dialogue.

### Focus Groups

To further understand students’ goal-setting, in October-November 2021, EB and CT held three hybrid focus groups. At the end of the second small group session, EB invited all students to voluntarily sign up for a focus group. In each group, two medical and one or two nursing students participated (N = 11), two to six weeks after participating in WIFI. Focus groups started with a reminder of the goal-setting assignment and the Westerveld criteria. Then, students were asked to describe the processes of setting their goals and prompted to elaborate on the sources of information they used. Discussion between students was stimulated to elicit interprofessional and interpersonal differences and similarities in the availability and use of these sources.

EB, CT, RK, and SB analyzed focus group data using a three-step deductive approach. We found four main groups of sources of information that informed students’ goal-setting process:

1. Experience in clinical rotations, including experiences with feedback, collaboration, patients, observations, or having no experience at all. Example: *“I did hear that I am often defensive and that that’s not the intention. But that I then indeed can have the tendency to explain myself and that it comes across as not accepting the feedback”. (Student 1.4)*
2. WIFI, including the WVF criteria, the principle descriptions, the giver and user roles. Example: *“Yes, so I just looked at what criteria indeed and also thought like oke, what do I think I can learn. And for me that was feedback receiver for sure, and in terms of criteria that I would think: oke what matches the best and for me that was Actionable.” (Student 2.1)*
3. Personal characteristics, including norms and values, character, self-efficacy, and interpretation of own strengths and weaknesses. Example: *“First I looked at the different elements: what do I find important and what do I have few experience with? And what do I appreciate in other people, that I don’t do that well yet and how can I make a learning goal out of it, to improve myself”. (Student 3.1)*
4. Outcome expectations, including expectations of colleagues, mentors, and assessors (power dynamics), of the practical workplaces, and of possibilities within their position as students. Example: *“Yes, that’s what I’ll be working on in the coming weeks, because I have to coach people and in that system also have supervision meetings. So that’s what I’ll be working on”. (Student 2.2)*

## Critical Reflection on Process

We implemented an interprofessional feedback intervention for medical and nursing students, based on the WVF [4]. At the end of the intervention, students set goals to improve their feedback dialogue skills in their next rotations, which we analyzed to understand their learning outcomes.

Regarding the submitted goals, students expressed intentions to improve as feedback information *givers* and mainly improve on feedback *actionability*, followed by *timely, responsiveness*, or *dialogical* form of their feedback dialogues. In contrast, their written goals mainly addressed the criterion *open and respectful*, particularly in terms of *giving* feedback information, and *assertiveness*. This emphasis on initiating feedback dialogues aligns with both our focus group finding, that students’ aims to overcome expected barriers to feedback were a main source in their goal-setting, and the literature, which widely reports students’ challenges in initiating dialogues [14,15,16]. We see a contrast between initiation goals and students wanting to improve on other aspects of feedback dialogue (giving, actionable feedback information). This might imply that learning to initiate a dialogue, and overcoming contextual and interpersonal barriers to this initiation, is at least prioritized before, and may even be conditional to, the development of other aspects of feedback dialogue. Thus, addressing this initiation aspect should be a priority in future adaptations of interprofessional feedback education.

We also found nursing students were significantly more likely to want to improve as feedback information givers than medical students. This may reflect nursing students’ ambitions to overcome the classical interprofessional power dynamics in health care [17], i.e. for them to feel more comfortable giving feedback information to physicians.

Finally, as students described the process of setting goals for their interprofessional feedback dialogues, they combined four main groups of sources of information in their narratives: *experience in workplace rotations, outcome expectations, interprofessional feedback education (using the Westerveld framework)*, and *personal characteristics*. These groups resonate with well-known influences on goal setting e.g., problems with current state, traits, and situational constraints [11,12]. Having students explicitly discuss and combine these groups of sources in education may support their interprofessional feedback goal setting.

Still, we need to address two important limitations of our evaluation. First, the goal setting assignment had a low response rate (27%). The assignment submission was anonymous and voluntary, because the coordinator did not want to force students to submit this personal information. Also, it was observed that more students had written down their personal learning goal, but just did not fill in the digital form. As these assignments were collected anonymously, we could not check to what extent the focus group students were representative of the whole student population. We do think selection bias might have taken place, with students that value interprofessional feedback dialogues being more inclined to join the focus groups.

Second for the student goals that were submitted, we found that these often lacked specificity. As goal-setting theory predicts low task performance when goals are unspecific [12], we may simply need to encourage specific goal-setting in training. But the teachers explained this with (medical) students being more focused on summative knowledge assessments compared to skills education like this innovation and might not have seen its value. Still, the lack of specificity could also be attributed to the complexity of the task students were setting goals for. For instance, many students mentioned power dynamics as a barrier to achieve their goal. However, when understood and explored not as a barrier, but as a goal in conflict with another goal (e.g., a feedback goal), students may be more able to deliberately choose to act on one goal or another in practice. Focus group students often mentioned multiple goals (e.g., wanting to be safe and liked in a learning environment *and* wanting to be an honest interprofessional communicator) [18,19]. Such seemingly compatible goals on a higher, more abstract, level can raise conflict on a lower, more specific, level of abstraction (e.g., wanting to keep a low profile or agree with an interprofessional senior colleague *and* wanting to speak up to them) [19]. Recognizing and incorporating these conflicting goals into interprofessional feedback education can help medical and nursing students navigate the complexities of interprofessional collaboration and address perceived barriers effectively.

In conclusion, our contribution to the improvement of interprofessional feedback education is twofold. First, we have showcased a way to use the WVF to train students for interprofessional feedback dialogues. Second, we provide a deeper understanding of students’ goal setting for their clinical interprofessional feedback dialogues, as they partake in such training. Furthermore, the challenge to initiate feedback dialogues may be conditional to, and therefore overshadow, other possible goal content. To better support students in interprofessional feedback dialogue education, they must be made aware of these challenges, supported in developing strategies to overcome them, and offered relevant information sources to discuss whilst setting learning goals.
